# Immunogenicity of a Killed Bivalent (O1 and O139) Whole Cell Oral Cholera Vaccine, Shanchol, in Haiti

**DOI:** 10.1371/journal.pntd.0002828

**Published:** 2014-05-01

**Authors:** Richelle C. Charles, Isabelle J. Hilaire, Leslie M. Mayo-Smith, Jessica E. Teng, J. Gregory Jerome, Molly F. Franke, Amit Saha, Yanan Yu, Paul Kováč, Stephen B. Calderwood, Edward T. Ryan, Regina C. LaRocque, Charles P. Almazor, Firdausi Qadri, Louise C. Ivers, Jason B. Harris

**Affiliations:** 1 Division of Infectious Diseases, Massachusetts General Hospital, Boston, Massachusetts, United States of America; 2 Department of Medicine, Harvard Medical School, Boston, Massachusetts, United States of America; 3 Partners In Health, Boston, Massachusetts, United States of America; 4 Division of Global Health Equity, Brigham and Women's Hospital, Boston, Massachusetts, United States of America; 5 Department of Global Health and Social Medicine, Harvard Medical School, Boston, Massachusetts, United States of America; 6 International Centre for Diarrhoeal Disease Research, Bangladesh (icddr,b), Dhaka, Bangladesh; 7 National Institute of Diabetes and Digestive and Kidney Diseases, Laboratory of Bioorganic Chemistry, National Institutes of Health, Bethesda, Maryland, United States of America; 8 Department of Microbiology and Immunobiology, Harvard Medical School, Boston, Massachusetts, United States of America; 9 Department of Immunology and Infectious Diseases, Harvard School of Public Health, Boston, Massachusetts, United States of America; 10 Department of Pediatrics, Harvard Medical School, Boston, Massachusetts, United States of America; Instituto Butantan, Brazil

## Abstract

**Background:**

Studies of the immunogenicity of the killed bivalent whole cell oral cholera vaccine, Shanchol, have been performed in historically cholera-endemic areas of Asia. There is a need to assess the immunogenicity of the vaccine in Haiti and other populations without historical exposure to *Vibrio cholerae*.

**Methodology/Principal Findings:**

We measured immune responses after administration of Shanchol, in 25 adults, 51 older children (6–17 years), and 47 younger children (1–5 years) in Haiti, where cholera was introduced in 2010. A≥4-fold increase in vibriocidal antibody titer against *V. cholerae* O1 Ogawa was observed in 91% of adults, 74% of older children, and 73% of younger children after two doses of Shanchol; similar responses were observed against the Inaba serotype. A≥2-fold increase in serum O-antigen specific polysaccharide IgA antibody levels against *V. cholerae* O1 Ogawa was observed in 59% of adults, 45% of older children, and 61% of younger children; similar responses were observed against the Inaba serotype. We compared immune responses in Haitian individuals with age- and blood group-matched individuals from Bangladesh, a historically cholera-endemic area. The geometric mean vibriocidal titers after the first dose of vaccine were lower in Haitian than in Bangladeshi vaccinees. However, the mean vibriocidal titers did not differ between the two groups after the second dose of the vaccine.

**Conclusions/Significance:**

A killed bivalent whole cell oral cholera vaccine, Shanchol, is highly immunogenic in Haitian adults and children. A two-dose regimen may be important in Haiti, and other populations lacking previous repeated exposures to *V. cholerae*.

## Author Summary

Studies evaluating the ability of the killed bivalent whole cell oral cholera vaccine, Shanchol, to elicit an immune response have been performed in historically cholera-endemic areas of Asia. There is a need to assess whether the vaccine is able to elicit an immune response in Haiti and other populations without historical exposure to cholera. In this study, we measure immune responses after administration of Shanchol, in 25 adults, 51 older children (6–17 years), and 47 younger children (1–5 years) in Haiti, where cholera was introduced in 2010. A killed bivalent whole cell oral cholera vaccine (Shanchol) is capable of inducing an immune response in adults and children living in Haiti. However, a two-dose regimen may be important in Haiti and other populations lacking historical exposure to cholera.

## Introduction

Cholera remains a public health problem for many of the world's poorest individuals. Approximately 3 million cases of diarrheal illness and 120,000 deaths are caused by *V. cholerae* annually [1]. Devastating epidemics occur when *V. cholerae* is introduced into an immunologically naive population that lacks access to safe water and sanitation. This occurred when a pandemic *V. cholerae* O1 strain was introduced into Haiti in 2010 [2], [3], resulting in 693,088 cases and 8474 reported deaths as of November 27, 2013 [4]. The increasing burden of endemic and epidemic cholera has led to recognition that new approaches to the control of cholera, including vaccination, are urgently needed [5], [6].

There are two currently licensed cholera vaccines. Both are oral killed whole cell vaccines that have demonstrated efficacy in preventing cholera in endemic settings. Dukoral (Crucell) is a whole cell recombinant cholera toxin B subunit vaccine (WC-rBS) which contains both the Inaba and Ogawa serotypes of *V. cholerae* O1, and recombinant cholera toxin B subunit (CTB). Shanchol (Shantha Biotechnics) is a bivalent whole cell vaccine which contains *V. cholerae* serogroups O1 and O139, but lacks CTB. Shanchol is less expensive than Dukoral and may be associated with longer lasting protection [7]–[10].

The World Health Organization (WHO) recommends that cholera vaccines be used in cholera-endemic settings [11]. However, the use of vaccination during epidemics remains controversial, and in 2010 the WHO position paper on cholera vaccination encouraged studies of the feasibility and impact of vaccination in the setting of ongoing outbreaks of cholera [11].

Recent pilot vaccination campaigns in Haiti, South Sudan, and Guinea have demonstrated the feasibility of reactive and/or preventive cholera vaccination [12]–[14]. In a pilot vaccination campaign in rural Haiti, Shanchol was distributed to 45,417 individuals in conjunction with health education messages regarding household water safety and sanitation. Despite logistical challenges in this setting, a vaccination coverage rate in excess of 75% was achieved [12], exceeding the 50% threshold associated with high levels of herd immunity [15]. Notably, 91% of vaccine recipients in the pilot campaign in Haiti received the recommended two doses of the vaccine [12].

While the immunogenicity of Shanchol has been demonstrated in South Asia [8], [16], no studies of the immunogenicity of this vaccine have yet been reported outside of historically cholera-endemic areas. Prior experience suggests that immunogenicity and efficacy of cholera vaccines in populations from historically cholera-endemic areas of Asia may not be extrapolated to populations from other geographic regions. For instance, a study conducted in Peru shortly after the introduction of *V. cholerae* in 1991 demonstrated that a third dose of the WC-rBS vaccine was required to provide a high rate of seroconversion and boost protective efficacy from 0% to 61% [17]. In contrast, a two-dose regimen of a similar vaccine was associated with 86% protection in Bangladesh [18].

In this study, we address a knowledge gap regarding the use of Shanchol in epidemic settings. To assess the immunogenicity of this vaccine in Haiti, we measured vibriocidal antibody responses, the best characterized immunologic correlate of protection against cholera [19], [20]. We also assessed IgA responses to the O-antigen specific polysaccharide (OSP), the primary determinant of lipopolysaccharide antigen specificity [21]. We included young children in our analysis, since they are disproportionately affected by cholera [22] and may mount less robust immune responses to cholera vaccination [7], [23], [24]. We also included a comparison of immune responses of Haitian vaccinees to Bangladeshi vaccinees to assess whether immune responses to Shanchol would differ in individuals from a historically cholera-endemic area compared to an area where cholera has recently been introduced.

## Methods

### Enrollment of study participants

Subjects were enrolled in St. Marc, Haiti, in April 2013. Subjects 1 year of age and older were eligible to participate. Exclusion criteria included pregnancy, acute medical illness, prior receipt of oral cholera vaccine, or a history of hospitalization for cholera. We also analyzed plasma samples obtained from Bangladeshi individuals who participated in a previously conducted study of the immunogenicity of Shanchol in Bangladesh [16]. The Bangladeshi individuals whose samples were utilized were selected at random from a larger cohort of vaccinees and then age-matched to Haitian vaccinees to within 1 year for young children and within 5 year for adults and blood-group matched for either O or non-O blood groups.

### Ethics statement

The studies were approved by the institutional review board of Partners HealthCare (Brigham and Women's Hospital and Massachusetts General Hospital), the Haitian National Ethics Committee, and the Ethical Review Committee of the International Centre for Diarrhoeal Disease Research in Dhaka, Bangladesh (icddr,b). Written informed consent was obtained from adult participants and from guardians of children.

### Immunization

Participants were administered two doses of Shanchol, given 14 days apart. Participants were monitored for 30 minutes after vaccination and were asked to return to the study site or to contact a study coordinator if they felt ill after receipt of the vaccine. Adverse events were evaluated and recorded by study physicians.

### Samples

Venous blood samples were obtained immediately prior to immunization (day 0) and seven days after each dose of vaccination (day 7 and day 21). Serum was stored at −80°C, and shipped to Massachusetts General Hospital. To avoid any laboratory-related biases, plasma from Bangladesh from a previously conducted study of the immunogenicity of Shanchol vaccine was stored, shipped, and analyzed at Massachusetts General Hospital concurrently with the Haitian samples [16].

### Immunologic assays

Vibriocidal antibody assays were performed as described previously [16], [25]. Target strains of *V. cholerae* O1 Inaba (T19479) and Ogawa (X25049), were incubated with heat inactivated serum and exogenous guinea pig complement. Vibriocidal titers were defined as the reciprocal of the highest serum dilution resulting in a 50% reduction in optical density (595 nm) compared to controls without serum. To account for inter-assay variation, results were normalized using high titer sera. Seroconversion was defined as a 4-fold or greater increase from the baseline vibriocidal titer after vaccination.

CTB and OSP responses were measured using a previously described ELISA [23], [26]. OSP plates were coated with 1 µg of conjugated OSP:BSA per milliliter of carbonate buffer (pH 9.6). CTB plates were coated with GM1 ganglioside followed by recombinant CTB (2.5 µg/ml). Sera (diluted 1∶25 for OSP, 1∶50 for CTB) was added to plates, and IgA responses were detected with goat anti-human IgA conjugated with horseradish peroxidase (Jackson ImmunoResearch). A 2-fold or greater rise in milliabsorbance units per second at day 7 or day 21 compared to day 0 was considered a significant response. Because Shanchol does not contain CTB, these responses were assessed to ensure that vibriocidal and OSP responses were not due to either intercurrent natural *V. cholerae* infection or non-specific immune activation.

### Statistical analysis

Statistical analyses were performed using STATA Version 9. Antibody titers were log_2_ transformed, and the log-transformed data were used for statistical analyses. The immunologic results were expressed as geometric means (GMT) and compared by a paired *t*-test for within group comparison and by the Kruskal-Wallis analysis of variance (ANOVA) and/or Student's *t*-test for between group comparisons as indicated. Analysis of proportions was performed using chi-square or the Fisher exact test as appropriate. For the matched comparison between the Haitian and Bangladeshi cohorts, subjects were matched by age (within one year for all children, and within 5 years for adults) and blood group; the differences between GMT were assessed using a paired t-test. The threshold for statistical significance was a two-tailed p value of <0.05.

## Results

### Study enrollment and participation

The study cohort consisted of 123 Haitian participants (Table 1), of whom 25 were adults (≥18 years), 51 were older children (6–17 years), and 47 were younger children (1–5 years).

**Table 1:** Demographic characteristics of Haitian study participants.

Characteristics	Total	Adults (≥18 yo)	Older children (6–17 yo)	Young Children (1–5 yo)
	*N* = 123	*N* = 25	*N* = 51	*N* = 47
**Mean Age, years (S.D.)** *	**16.6 (15)**	**32.7 (11)**	**11.3 (4)**	**3.1 (1)**
**Percent Female**	**63**	**80**	**61**	**55**
**Percent Blood Type O**	**51**	**44**	**59**	**47**

*standard deviation.

**Figure 1:**
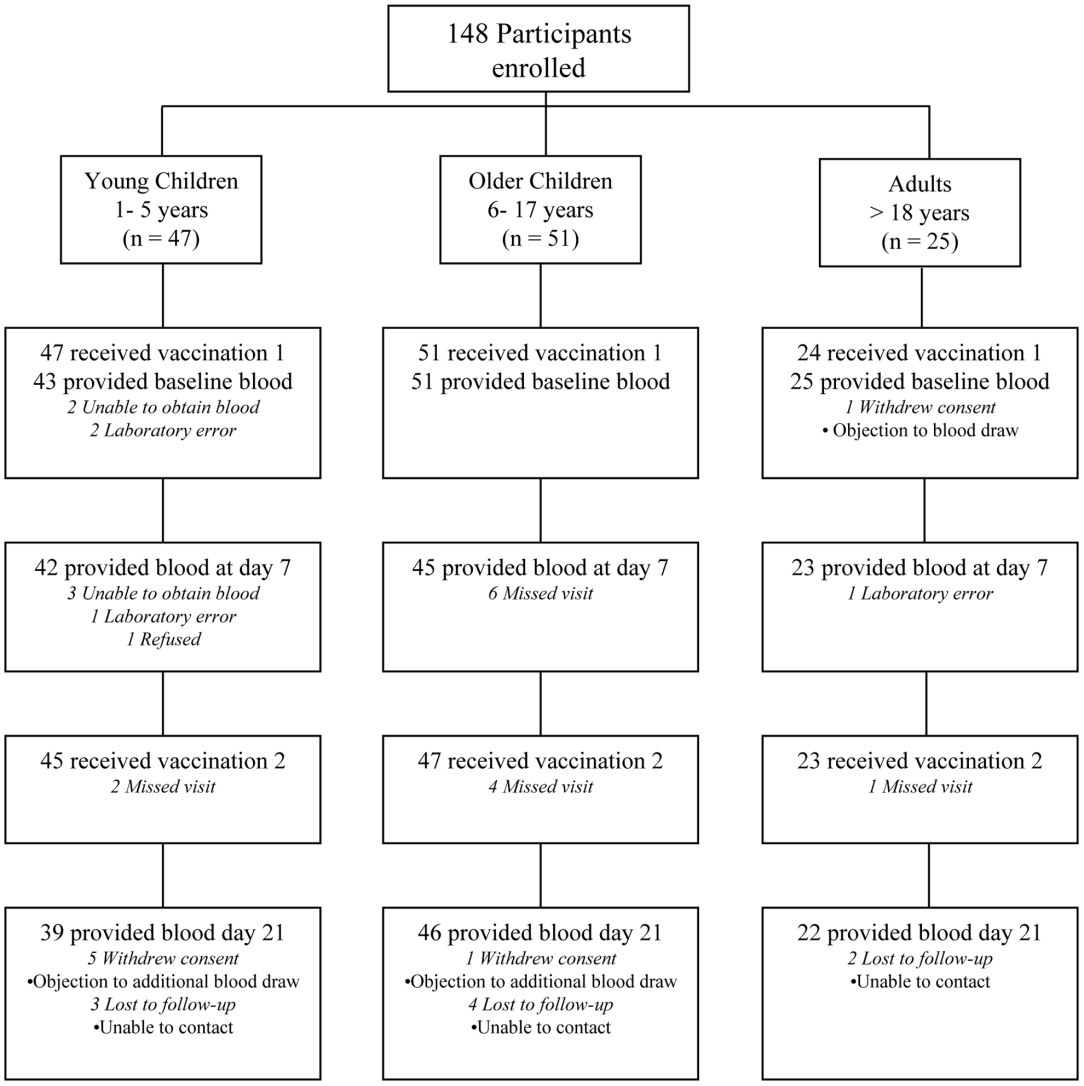
Enrollment and follow-up of study participants.

A flowchart of participants through the study is provided in Figure 1. Of the 123 participants, 115 (93%) received both doses of the vaccine. No adverse events related to vaccination were reported among participants.

### Vibriocidal antibody responses

**Figure 2:**
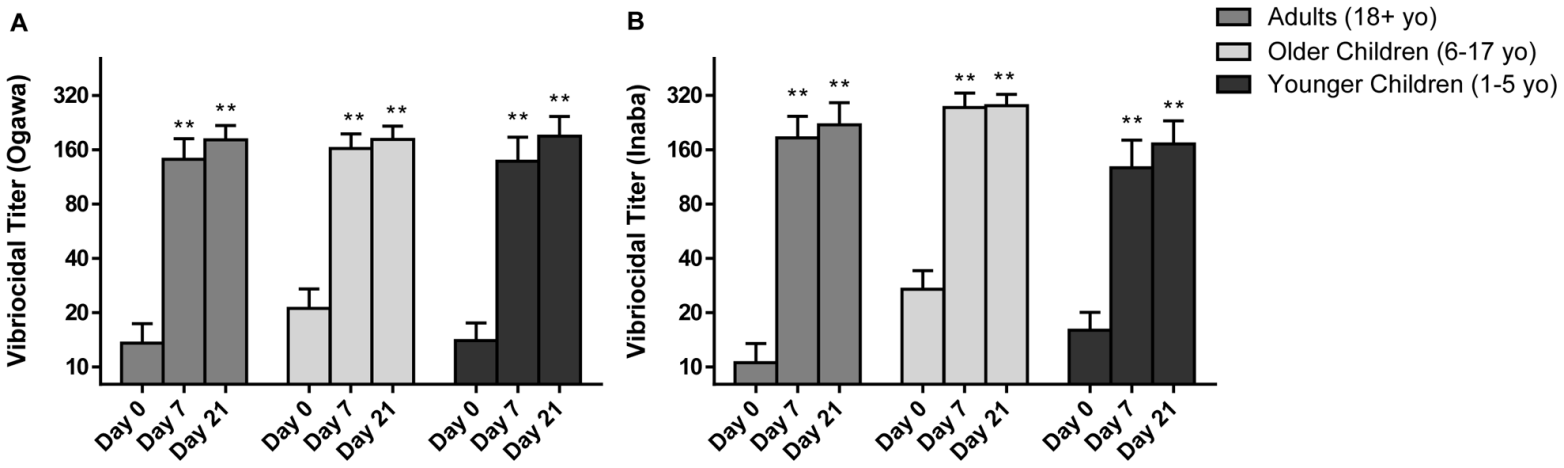
Vibriocidal responses. Geometric mean titer (+SEM) of vibriocidal responses to *V. cholerae* O1 Ogawa (A) and Inaba (B) by age group at baseline (day 0) and 7 days after each immunization (day 7 and day 21). Statistically significant differences relative to baseline are indicated (**  =  <0.001).

Figure 2 shows the vibriocidal antibody responses. The baseline GMT to *V. cholerae* O1 Ogawa was 14, 21, and 14 for adults, older children, and young children, respectively; and for Inaba the baseline GMT was 11, 27, and 16 for each age cohort. There was a statistically significant difference in baseline GMT between adults and older children (p = 0.02) to *V. cholerae* O1 Inaba, but there was no difference in baseline GMT among age cohorts for Ogawa. Overall, 34% of participants had a baseline vibriocidal titer ≥80 to Ogawa and/or Inaba; 14% of participants had a GMT ≥320 and 7% of participants had a GMT ≥640. This suggests that many individuals had been recently exposed to *V. cholerae*. However, the proportion of individuals with a baseline vibriocidal titer ≥80 did not differ significantly between age cohorts.

Each of the age cohorts had a robust vibriocidal antibody response by day 7, after a single dose of Shanchol (p<0.0001 for all cohorts) with a geometric mean fold rise (GMF) of 11, 9, and 11 in adults, older children, and young children to *V. cholerae* O1 Ogawa, and 19, 11, and 9 to Inaba. Vibriocidal GMTs increased further in each of the age cohorts after the second dose of the vaccine. Although these increases between day 7 and day 21 were not statistically, significant, the GMT at day 21 was significantly increased compared to baseline values in all age cohorts (Ogawa GMF: 13, 9, 12 and Inaba GMF: 19, 10, 10 for adults, older children, and young children) (p<0.0001).

The majority of adults, older and younger children demonstrated vibriocidal antibody seroconversion against both serotypes (Table 2). Approximately half of the individuals who failed to seroconvert after vaccination had high baseline titers suggestive of recent exposure. Of the 22 vaccinees who did not seroconvert to Ogawa, 15 (68%) had a baseline vibriocidal titer of ≥80, and of the 15 vaccinees who did not seroconvert to Inaba, 8 (53%) had a baseline titer ≥80.

**Table 2:** Seroconversion rates among Haitians receiving two doses of Shanchol.*

	Adults (≥18 yo)	Older children (6–17 yo)	Young Children (1–5 yo)
	*N = 22*	*N = 42*	*N = 33*
**Vibriocidal (Ogawa)**	**91%**	**74%**	**73%**
**Vibriocidal (Inaba)**	**91%**	**86%**	**79%**
**OSP IgA (Ogawa)**	**59%**	**45%**	**61%**
**OSP IgA (Inaba)**	**50%**	**55%**	**55%**

*vibriocidal ≥4-fold increase, OSP (O-Specific Polysaccharide) ≥2-fold increase in kinetic ELISA.

### *V. cholerae*-specific serum antibody responses

**Figure 3:**
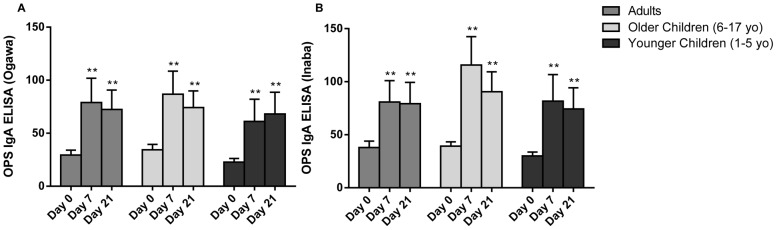
OSP-specific IgA responses. OSP-specific IgA responses to *V. cholerae* O1 Ogawa (A) and Inaba (B) by age group at baseline (day 0) and 7 days after each immunization (day 7 and day 21). Statistically significant differences relative to baseline are indicated (**  =  <0.001).

Vaccinees across all age groups developed significant OSP-specific IgA serum responses at day 7 and day 21 (Figure 3). Rates of seroconversion, as defined as a 2-fold rise in antibody level, were comparable across age groups for OSP of Ogawa and Inaba (Table 2). We also evaluated CTB IgG and IgA serum responses on a subset of patients. As expected, there was no increase in CTB responses after immunization (data not shown).

### Children younger than 2 years

Because children younger than two years have diminished responses to T-cell independent vaccine antigens, we compared children younger than 2 years of age (N = 8) with children between 2 and 5 years (N = 35). The very young children (<2 years) had a lower vibriocidal GMT on day 7 compared to the 2–5 years old children (Ogawa GMT 40 vs. 177, p = 0.07; Inaba GMT 20 vs 184, p = 0.02). However, after two doses of the vaccine there was no statistically significant difference in the vibriocidal GMT between 1 year olds and 2–5 year olds (Ogawa GMT 226 vs 183 p = 0.76; Inaba GMT 80 vs. 200 p = 0.26).

### Relationship of blood group with post-vaccination titers

**Figure 4:**
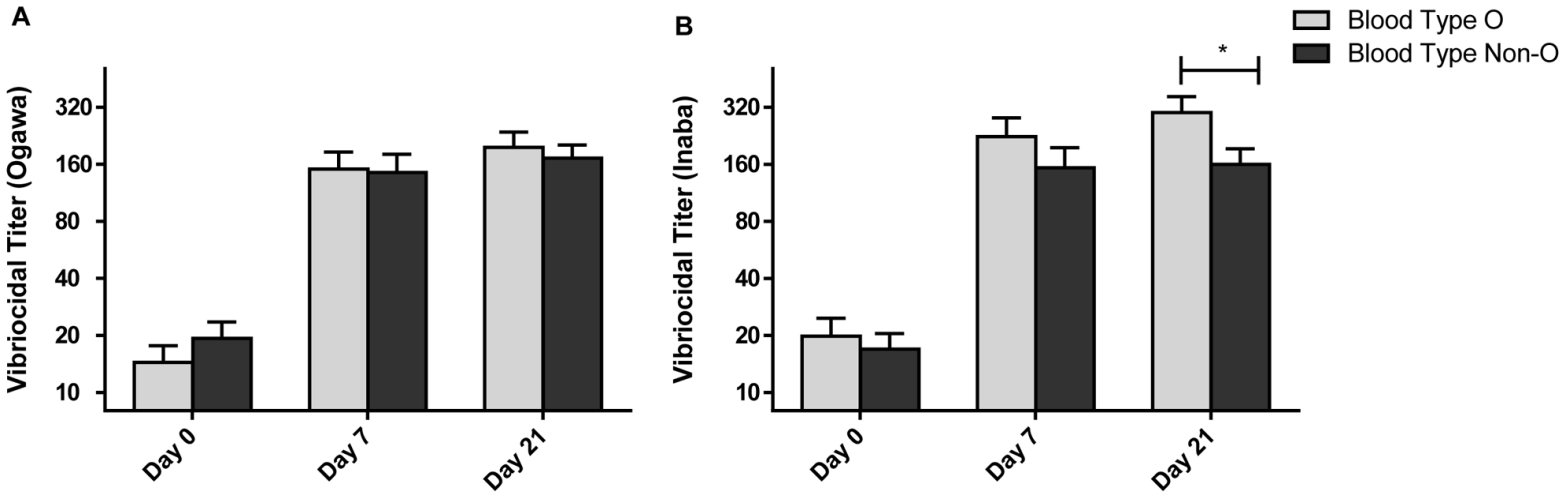
Vibriocidal responses in O and Non-O blood groups. Geometric mean titer (+SEM) of vibriocidal responses to *V. cholerae* O1 Ogawa (A) and Inaba (B) by blood group at baseline (day 0) and 7 days after each immunization (day 7 and day 21). Statistically significant differences across blood groups are indicated (*  =  <0.05).

Overall, 63 individuals were blood type O (51.2%). Individuals with blood type O had a greater vibriocidal GMT against serotype Inaba after two doses of vaccine compared to study participants with non-O blood types (GMT = 300 for group O, GMT = 160 for non-O p = 0.02), but there was no significant difference in the vibriocidal response to the Ogawa serotype (Figure 4). There were no significant differences in magnitude in OSP-specific IgA responses between blood groups (data not shown).

### Comparison of immune responses in Haitian and Bangladeshi vaccinees

**Figure 5:**
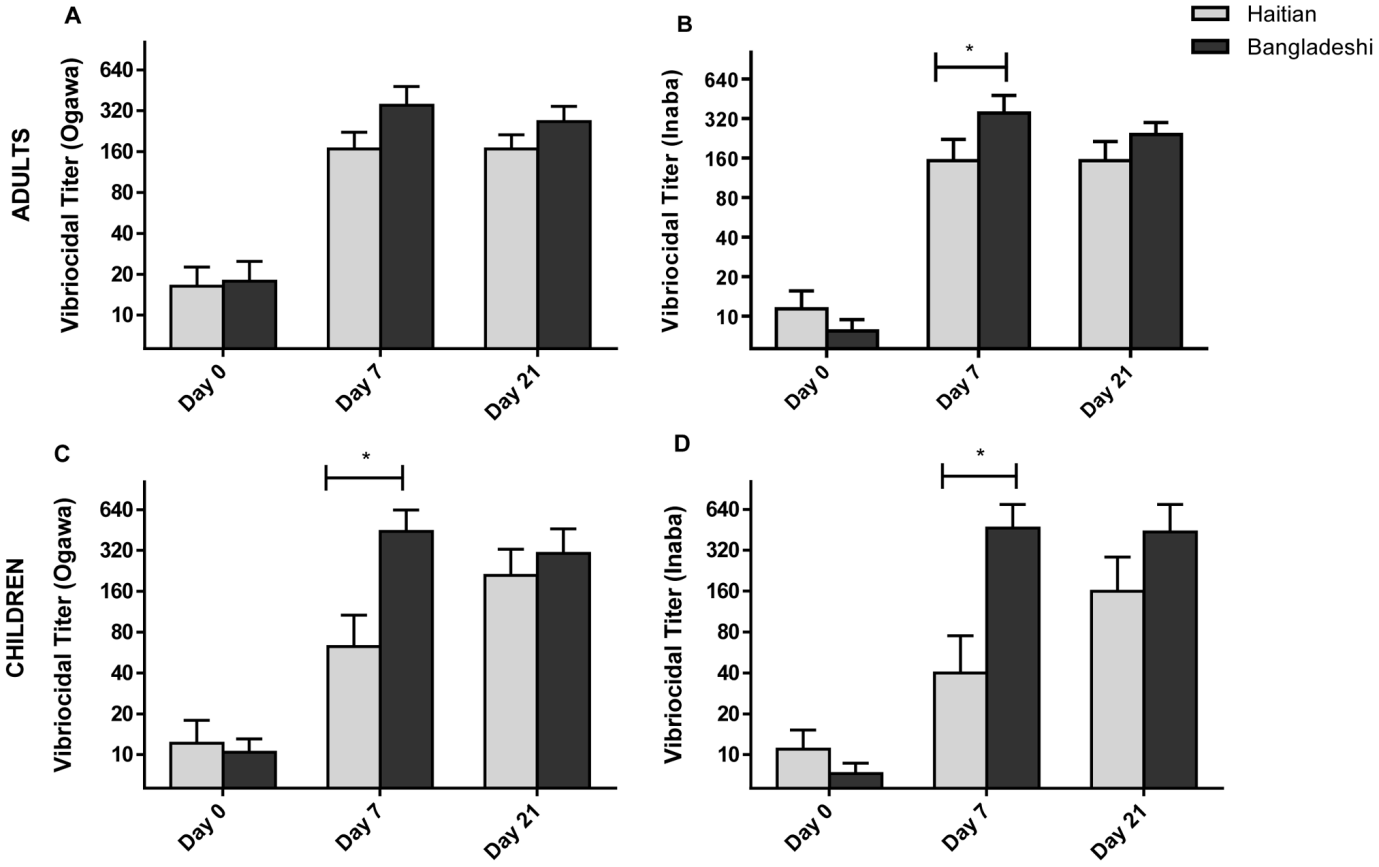
Vibriocidal responses in Haitian versus Bangladeshi vaccinees. Geometric mean titer (+SEM) of vibriocidal responses to *V. cholerae* O1 Ogawa and Inaba at baseline (day 0) and 7 days after each immunization (day 7 and day 21) in adults (A and B) and children (C and D). Statistically significant differences across countries at a given day are indicated (*  =  <0.05).

We compared immune responses in a subset of Haitian vaccinees with age- and blood group-matched vaccinees from Bangladesh (Figure 5). The cohort from Bangladesh consisted of 17 adults and 20 young children aged 1–5 years. Notably, half of the young children in the matched cohorts were younger than 2 years old, which skewed the pool of young children in the matched analysis toward a younger average age (2.3 years in the matched cohort, 3.1 years in the larger Haitian cohort).

Even though cholera has been endemic in Bangladesh, and only recently introduced in Haiti, the baseline GMT vibriocidal antibody titers and proportion of individuals with baseline vibriocidal titers ≥80 were comparable between the two matched cohorts.

Among the matched cohorts, the adult Bangladeshi vaccine recipients had higher vibriocidal GMT after the first dose of vaccine compared to adult Haitian vaccinees (Figure 5A and 5B). This was statistically significant for the Inaba serotype (Inaba GMT in Haitians  = 153 and Bangladeshis  = 351, p = 0.03). However, this difference was diminished and no longer significant after a second dose of the vaccine (Inaba GMT for Haitians  = 153 and Bangladeshis  = 243, p = 0.20).

The difference between matched cohorts at day 7 was more pronounced for young children (Figure 5C and 5D). The vibriocidal GMT to *V. cholerae* O1 Ogawa at day 7 was 63 for Haitian young children compared to 443 for Bangladeshi young children (p = 0.002); and 40 for Haitians and 465 for Bangladeshi to *V. cholerae* O1 Inaba (p = 0.004). Again, the differences are diminished and no longer statistically significant after a second dose of vaccine (Ogawa GMT in Haitian young children  = 209 and Bangladeshi young children  = 303, p = 0.57; Inaba GMT for Haitians  = 160 and Bangladeshis  = 435, p  = 0.31).

## Discussion

To our knowledge, this is the first report of the immunogenicity of the killed bivalent whole cell cholera vaccine, Shanchol, outside of historically cholera-endemic areas of Asia. We found that Shanchol was immunogenic across all age groups in Haiti, from adults to very young children. Given the ongoing transmission of *V. cholerae* O1 in Haiti, these findings provide additional evidence to suggest that expansion of cholera vaccination programs would aid cholera control efforts in Haiti.

The results of this study are notable because *V. cholerae* was only recently introduced in Haiti. Previous studies of the immunogenicity and efficacy of Shanchol have been conducted in the historically cholera-endemic Ganges delta area of South Asia [7]–[10], [16]. Because levels of previous or repeated exposures to *V. cholerae* may dictate the magnitude of vaccine responses, it was unclear whether similar immune responses would be seen in the Haitian population. In addition, recent evidence suggests that genetic factors influencing the innate immune response to *V. cholerae* may differ in individuals of Bengali ethnic heritage, providing another possible reason for differing immune response to cholera vaccines between different populations [27].

In fact, the results of our direct age- and blood group- matched comparisons suggest the immunogenicity of Shanchol is comparable in Bangladesh and Haiti, at least when administered in a two-dose regimen. Vibriocidal antibody titers are considered an important marker for assessing the immunologic response to cholera vaccines [28], [29], and we found the proportion of Haitian vaccinees who demonstrated vibriocidal seroconversion approached or exceeded 75% across all age cohorts. This compares favorably with the seroconversion rates seen in Bangladeshi vaccinees [16]. In addition, the final magnitude of the vibriocidal antibody response after two doses of the vaccine was comparable in Haitian and Bangladeshi individuals.

The major difference between the Bangladeshi and Haitian study populations were that immune responses were of a greater magnitude after the first dose of Shanchol among Bangladeshis. We believe this difference is most likely explained by a higher level of immunologic priming from previous or repeated exposure to *V. cholerae* in the Bangladeshi vaccinees. The observation that baseline vibriocidal titers were similar in both groups seems counter to this hypothesis, but we and others have previously shown that vibriocidal antibodies are a relatively short-term immunologic marker of past-exposure [25], [30]. Thus it is possible, that longer lasting memory B cell responses following natural *V. cholerae* infection may mediate anamnestic responses to vaccines in historically cholera-endemic areas. An alternative possibility is that this difference is due to other genetic or environmental differences, though this seems less plausible as the differences are mitigated (rather than accentuated) by a second dose of the vaccine. Regardless, our findings suggest that the efficacy of single dose oral cholera vaccine may differ in an epidemic versus a historically cholera-endemic setting.

This report also includes the first analysis of O-specific polysaccharide responses to *V. cholerae* outside of Asia. The vibriocidal response is largely thought to be comprised of IgM targeting *V. cholerae* lipopolysaccharide (LPS) [31], [32]. Protective immunity against *V. cholerae* is serogroup-specific, and serogroup specificity is defined by OSP. OSP is a T cell independent antigen, and young children are not typically able to mount immune responses against such antigens. This is unfortunate since, during a cholera epidemic among an immunologically-naïve population, cholera afflicts children and adults equally, and in endemic populations, children bear a large burden of cholera. In our analysis, however, we found that young children were able to mount serum IgA OSP responses that were comparable to those induced in older children and adults. This is in contrast to serum IgA OSP responses observed with administration of Dukoral to children in Bangladesh where only children over the age of 5 had a significant OSP response to Dukoral [23]. This difference may be due to the lower concentration of LPS present in Dukoral compared to Shanchol, or due to the immunodulatory effects of CTB present in Dukoral vaccine [33]. Interestingly, despite the observation that a second dose of Shanchol boosted vibriocidal responses, no additional boosting of OSP serum IgA responses was observed following a second dose in younger and older Haitian children. The significance of these observations is uncertain.

The ABO blood group has been implicated in the susceptibility and severity of cholera [34]–[36], as well as the immunogenicity and efficacy of oral cholera vaccines, with higher responses and efficacy generally seen in the blood group O population which is at greater risk of severe cholera [37], [38]. Our study also showed increased vibriocidal antibody responses to Shanchol in blood group O individuals, although this was only seen for Inaba serotype after two doses of the vaccine.

There are some important limitations of our study. While the vibriocidal antibody response is the historical immunologic benchmark for cholera vaccines [28], it is at best an indirect marker of protection as the protection afforded by cholera vaccines outlasts a measurable vibriocidal response by several years [39]. Thus, while immunologic results from our study support the expansion of vaccination efforts, epidemiologic data on vaccine efficacy is still important. In addition, our study design was not a placebo-controlled trial of Shanchol since such as design would not be acceptable in an epidemic setting. However, we believe our immunologic results clearly demonstrate vaccine-specific responses, given the rise in both vibriocidal and OSP-IgA antibodies without concurrent reactivity to CTB, which is not present in the Shanchol vaccine.

In summary, we demonstrated that Haitian adults and children had robust immune responses to the killed whole cell bivalent oral cholera vaccine Shanchol. This provides evidence that the vaccine is immunogenic even in areas where cholera has recently been introduced, and contributes to the body of evidence favoring the use of oral cholera vaccines as a component of comprehensive cholera control in epidemic settings in immunologically naïve populations.
